# Ocular surface and tear film changes in workers exposed to organic solvents used in the dry-cleaning industry

**DOI:** 10.1371/journal.pone.0226042

**Published:** 2019-12-09

**Authors:** Ingrid Astrid Jiménez Barbosa, Martha Fabiola Rodríguez Alvarez, Gerardo Andrés Dussán Torres, Sieu K. Khuu

**Affiliations:** 1 Health and Sciences Faculty, Optometry Program, University of La Salle, Bogotá, Colombia; 2 School of Optometry and Vision Science, The University of New South Wales, Sydney, Australia; UNSW, AUSTRALIA

## Abstract

Workers in the dry-cleaning industry are exposed to organic solvents that may cause eye irritation and tear film changes. **Objective** To quantify changes in the ocular surface and tear film in dry cleaners exposed to organic solvents and associate these changes with ocular irritation as reported in a symptom questionnaire for dry eye diagnosis. **Methods** This was a case and control study in which the characteristics and eye-irritation symptoms were compared between two groups of 62 participants that were either exposed or not exposed to organic solvents. A general optometric examination and the following test were performed: lipid interferometry, Lissamine Green Stain, tear breakup time, Schirmer I, conjunctival impression cytology and the Donate dry eye symptoms questionnaire. **Results** Sixty-five percent of exposed workers obtained a higher score than 13 on the Donate dry eye symptoms questionnaire which indicated the presence of more irritation symptoms than those in the non- exposed group. A Chi-square analysis indicated the exposed group reported significantly higher incidences (*P <0*.*005)* for eye irritation symptoms of sandy sensation; tearing eyes sensation; foreign body sensation; tearing; dry eye; dryness; eyestrain and heavy eyelids. A Mann Whitney-U indicated greater severity only for symptoms relating to dry eye; sandy sensation; foreign body sensation, tearing; tearing eyes and dryness. There was a statistically significant difference (*P* <0.05) for Schirmer I; tear break up time; and the ocular surface assessed with Lissamine green staining and conjunctival impression cytology between groups. A reduction in the thickness of the lipid layer in the exposed group compared to the non-exposed group was observed. Surprisingly, clinical test outcomes were not significantly correlated with dry eye symptoms nor years of exposure. **Conclusion** Workers in the dry-cleaning industry exposed to organic solvents are associated with changes in ocular surface and tear film generating irritation symptoms commonly present in evaporative dry eye.

## Introduction

Workers in the dry cleaning industry are commonly exposed to different occupational hazards and chemicals that may affect their general and, in particular, eye health [1]. Principle among which is the exposure to handling chemicals such as ethylene perchlorate, tetrachloro ethylene, Exxon, and some bleaches and soaps. These highly volatile lipophilic chemicals are organic solvents and are widely used in the industry and in household products as a means of dissolving fats/lipids from garments and materials. Organic solvents typically enter the body through the respiratory system, skin or mucosa and are known to cause neurotoxicity and adversely affect the central and peripheral nervous system [2,3].

Sensory detection of organic solvents by the individual is usually through the olfactory (smell) and taste along with the trigeminal somatosensory systems (irritation). At high chemical levels, exposure to organic solvents leads to unpleasant odor perception and cutaneous irritation (particularly the eye and skin), but the level of irritation varies according to the type of compound, concentration, time of exposure, as well as the type of response action of each person [4,5]. Importantly, sensory irritation is considered a clinical sign and the effect of organic solvent exposure on sensory assault (particularly exposure limits that result in nasal and somatosensory irritation) must be considered in the regulation of their use and risk prevention plans [6].

Organic solvent exposure can directly activate the somatosensory trigeminal system through the process of “chemesthesis”. Chemical agents induce sensations of burning, stinging, irritation or pain in the eyes and upper respiratory through the stimulation of the transient receptor potential TRP in the sensory nerve fibers [7,8]. Furthermore, chemical damage to mucous epithelium can release intracellular chemical and inflammatory mediators such as cytokines, bradykinin, histamine, and prostaglandins are involved in the activation of two types of both vanilloid 1 (TRPV1) and ankyrin 1 (TRPA1) which are associated in the translation of the sensation of pain and inflammation [6–9].

Though organic solvent exposure generally affects the nervous system function, commonly reported symptoms by dry cleaners and painters are associated with vision and reports of dry eye and eye irritation [10,11]. Given the chemical composition of organic solvents, exposure to them might cause disruption to the lipid layer of the tear film leading to increased rates of dry eye and eye irritation [12]. Our previous preliminary work has shown that exposure to organic solvents can lead to a decrease in the stability of the tear film and changes in the conjunctival epithelium (as measured using tear break up times and conjunctival impression cytology), and ocular symptoms such as dryness and grittiness [13,14]. The decrease in the stability of the tear film reflects a change in the lipid layer (due to the lipophilic nature of organic solvents), which may lead to the thinning of the tear film and or the stimulation of the sensory neurons of the ocular surface, which may cause eye irritation [15]. The instability of the tear film might eventually lead to pathological changes such as the decrease in goblet cells, intercellular connections, and disruption of cell membranes and release of inflammatory mediators [15,16]. In agreement, our previous work have found a decreased quantity of goblet cells and increased degree of squamous metaplasia in the bulbar conjunctiva in chronic exposure of organic solvents, suggesting a direct action of the organic solvents on the tear film and/or the ocular surface epithelium [14].

While, the effect of organic solvents on the stability of the tear film is not well understood, exposure to occupational levels of organic solvents has been shown to be associated with ocular irritation and dry eye symptoms [17,18]. Of note, and is the focus of the present study, is whether and how dry eye symptoms might be associated with changes in the tear film as measured by tear evaporation, hypo-secretion, but also chemical damage to epithelium or direct stimulation of the trigeminal nerve. This latter cause may be further exacerbated in cases where an inflammatory response is generated. Importantly, establishing whether a relationship exists between skin irritation and eye pain symptoms and damage to the ocular surface and or tear film, provides a means of understanding the direct consequences of chronic exposure to chemicals, and whether this is associated with somatosensory nervous system damage.

Whilst dry eye questionnaires such as Ocular surface disease Index (OSDI), The Dry Eye and McMonnies are frequently used in clinical and epidemiologic research, these questionnaires primarily focus on quality of life items and provide little indication of dry eye symptoms [19]. To address this issue, we used the Donate questionnaire, which is a questionnaire developed to specifically provide an indication of the level of various dry eye symptoms [20]). In the present study, the outcomes of the Donate questionnaire were compared with ocular surface characteristics such as the tear film, conjunctival hyperemia, conjunctiva or cornea damage evidenced by Lissamine green stain or conjunctival impression cytology. These biomarkers have been well documented to be associated with eye irritation and greater insight into the potential assault of organic solvent exposure on the ocular surface than previous studies that have simply sought to relate sensory perception change and exposure level [21–23].

## Methods

### Subjects

The study was a descriptive cross-sectional in design. The sample size of the study was 62 workers (exposed group) from fifteen independent dry-cleaning establishments located across Bogota Colombia and 62 subjects as normal (non-exposed group). This sample size (conforming to a power of 80% and Type I error probability of 5%) was determined using the PASS software based on the means for dry cleaners (76, SD±2.71), and for non-dry-cleaners (52, SD±1.52) on the Donate questionnaire from a previous study [24] that investigated dry eye and irritation symptoms.

The inclusion criteria for the cases were 18 to 40 years old, more than six months working at the dry-cleaning industry. Previous studies have measured the concentration levels of the organic solvent perchloroethylene in the environment and in alveolar air samples, which are considerably higher compared to non-dry-cleaning workplaces [25]. While measuring concentration levels of organic solvents were beyond the scope of the present study, a risk assessment of the workplaces was conducted in accordance with the Occupational Health, Security and Safety OHSAS 180001 (BS OHSAS 18001 2004) international guidelines [26– 28]. This assessment placed the level of risk and harm from chemical substances typically used in the dry-cleaning industry as “medium”, which requires continual monitoring. Finally, the dry-cleaning workplace is highly regulated by Colombian law [26] which requires mechanical ventilation to ensure normal workplace temperatures and good airflow. In addition, the city of Bogotá, where these laundries were located, has an average temperature between 8 and 19° C. Therefore, although there might be a moderate increase in temperature in the ironing area, but this is not excessive and should not exceed 25° C at workstations.

The exclusion criteria were any anterior segment infection, contact lenses users and any medication. For the control group, there was an additional criterion, these participants did not work or live close to the dry-cleaning establishment. Both case and control groups lived in the same city and were selected so that they had the same educational and socio economical level as the cases group and were matched for age. For both groups the recruitment process was the same, flyers were distributed in the main neighborhoods where significant numbers of dry-cleaning establishments were located; respondents were offered the chance to get gift cards to compensate for their time for participation.

The research was approved by the Ethics Committee of the Health and Science Faculty at the University of La Salle, and each participant gave their signed and informed consent. The study adhered to the tenets of the declaration of Helsinki.

### Procedures

A general optometric examination was performed to satisfy the inclusion and exclusion criteria, also the following clinical test were applied: evaluation of hyperemia using a slit lamp and the CCLRU grading scale, interferometry of the lipid layer; Lissamine green stain; Tear break up Time (TBUT); Schirmer I; conjunctival impression cytology and the Donate questionnaire for the diagnostic of dry eye symptoms. All these tests were selected according to the recommendations of the Dry Eye Work Shop [19,29], that such tests are appropriate to evaluate the tear film and the ocular surface in case of dry eye. We used standard protocols for the test described above and they were all performed by experienced and trained optometrists who were blind to whether the participant were cases or controls. Specific test details are given below. All tests were performed the same day during working hours, at the Optometry Clinic of La Salle university that was close by the dry-cleaning businesses. We confirmed with the occupational health and safety officer of the businesses that workers have been working all days of the week and the examination was performed at the end of that week considering the accumulative effect of the organic solvents in the worker’s body and working clothes.

#### Donate questionnaire

As mentioned, dry eye symptoms were evaluated by using the validated questionnaire of Donate, et al. (2002) [20]. This involves the subjective judgment of 22 eye symptoms which are distributed in this way: a) Symptoms related to lacrimal insufficiency such as red eye, eye discharge, dry eye, foreign body sensation, sandy sensation, itching, tearing, burning sensation, light sensitivity, eye strain. b) Symptoms related to palpebral pathology such as: swollen eyelids, crusts and scales on the eyelids, sticky eyes when you woke up, tearing eyes sensation, blurred vision that recovers with blinking, eye fatigue, and heavy eyelids sensation [20]. Some of these symptoms were surveyed twice using similar words that describe them in order to confirm agreement between subject response. This questionnaire has been reported to have 76% sensitivity and 71.4% specificity when compared to the Schirmer test which is considered as the gold standard [20].

The objective of the questionnaire and a brief description of each symptom was explained to each participant prior to testing, and they chose their response to each question using a Likert scale that varied between 0 to 4 (0 = without symptoms, 1 = symptom sometimes appears, 2 = symptom appears but does not represent a discomfort, 3 = symptom frequently appears, represents a discomfort and does not interferes in daily activities, 4 = symptom frequently appears, represents a discomfort and interferes in daily activities) to classify the subjective intensity of the symptom during the last week. For the presumptive diagnosis of dry eye, the reference score was 13 or greater.

#### Tear film lipid layer interferometry

The tear film lipid layer interferometry analysis was done using the Polaris device (Polaris team, Bon ©) and followed the protocol established with the Tearscope (Keeler ©), in which generated color patterns allowed quantification of the lipid content of tears. The interpretation was performed based on the methods described by Guillon [30], presented in Table 1:

**Table 1:** Interferencial lipid patterns (Guillón 1998), colour, appearance and estimated thickness.

Lipid layer pattern	Color	Appearance	Estimated thickness (nm)
Absent	Grey to White (30–60 nm)	The lipid layer is not visible	0–13
Open gray marble		Similar aspect to gray marble, poorly defined; often only visible after blinking	13–50
Closed marble		Similar aspect to gray marble well defined with a closed net	13–50
Fluid (wavy)	Yellow Grey (75 nm)	Changing wave type pattern	50–70
Amorphous	Yellow (90 nm)	Blue-white appearance without noticeable features	80–90
Normal colored	Yellow / Brown (105Nm) Brown / Yellow (120 nm) Brown (135nm) Brown / Blue (150nm) Blue (180nm)	Appearance of color fringing. Color changes are gradual	90–180 Variable
Anomalous Colored		Discrete fringe areas highly variable in color; quick color changes in a small area	

#### Lissamine green staining

This test was applied (1% drop) to the lower fornix of each eye, and a slit lamp with a neutral density filter and 10x magnification was used to evaluate the eye. Stained areas were counted and graded according to the Oxford classification [19,29]. Reference values are grade 0: 0–9 points of Lissamine green in the temporal and nasal bulbar conjunctiva, grade I: 10 to 32 points, grade II: 33 to 100 points, grade III: higher than 100 points, and grade IV greater than grade III.

#### Schirmer test I

This test was performed using special filter paper (Whatmann No: 41), that was positioned laterally on the side of the lower eyelids for 5 minutes. This was done without anesthetic and blinking. Reference values considered normal: greater than or equal to 10 mm / 5 minutes [19,29].

#### Tear Break up Test (TBUT)

TBUT was assessed through staining of the ocular surface and evaluated using sodium fluorescein strips of paper and a slit lamp with a cobalt blue filter. The time taken for the tear film to rupture was recorded. Reference value considered normal: average greater than or equal to 5 seconds [19,29].

#### Conjunctival impression cytology

It was assessed using a membrane of cellulose acetate esters HAWPO4700, pore 0,45μm (Millipore Corporation, Billerica). A strip of paper was located in the temporal and nasal bulbar conjunctiva after the application of topical anesthesia. The membranes were fixed with cell fixative (Fixcell ®) and colored with PAS-hematoxylin [31]. Each conjunctival impression cytology was graded according to the classification of Murube and Rivas [32]. Reference values were: Grade 0 and 1: normal; the presence of squamous metaplasia was considered from grade 2.

### Statistical analysis

Data normality for tear film measures was established using the *Shapiro Wilk test*, which found that the data were normally distributed. These variables were described with the mean and standard deviation. For bivariate analysis of the questionnaire, data *Mann Whitney-U test* was used. Results for impression cytology, Lissamine green staining and tear film lipid layer interferometry were described using percentage and a *Chi-square test* was applied to evaluate statistical significance.

## Results

### Demographics

The age of the exposed dry-cleaner group was 35 years (SD ± 9). Women comprised of 61.7% of the entire group. 47.7 percent of the exposed group worked in the industry for more than 5 years, 9.7% worked between 3 to 4 years, and 29.5% between 1 to 2 years and 13.1% less than 1 year. The daily exposure to organic solvents was 8 to 12 hours a day. Sixty-seven percent of the workers were involved in ironing, and 33% worked in other positions in the dry cleaning. The age of the non-exposed group was 36 (SD ± 6), and 60.86% were women, (see Table 2).

**Table 2:** Demographic information participants.

Demographic Characteristic	Dry- Cleaning group n = 62	Non- exposed group n = 62
**Age**	35 years (SD ± 9)	36 (SD ± 6)
**Gender**	Female 61.7% Male 38.3%	Female 60.86% Male 39.14%
**Years working in dry cleaning**	More 5 years 47.7% 3–4 years 9.7% 1–2 years 29.5% Less than 1 year 13.1%	None
**Hours working per day at the dry cleaning**	8 to 9 hrs	None
**Working positions at the dry cleaning**	Ironing 67% Prewash 5% Washing 15% Quality control 10% Administration 3%	None

### Dry eye symptoms questionnaire

**Fig 1:**
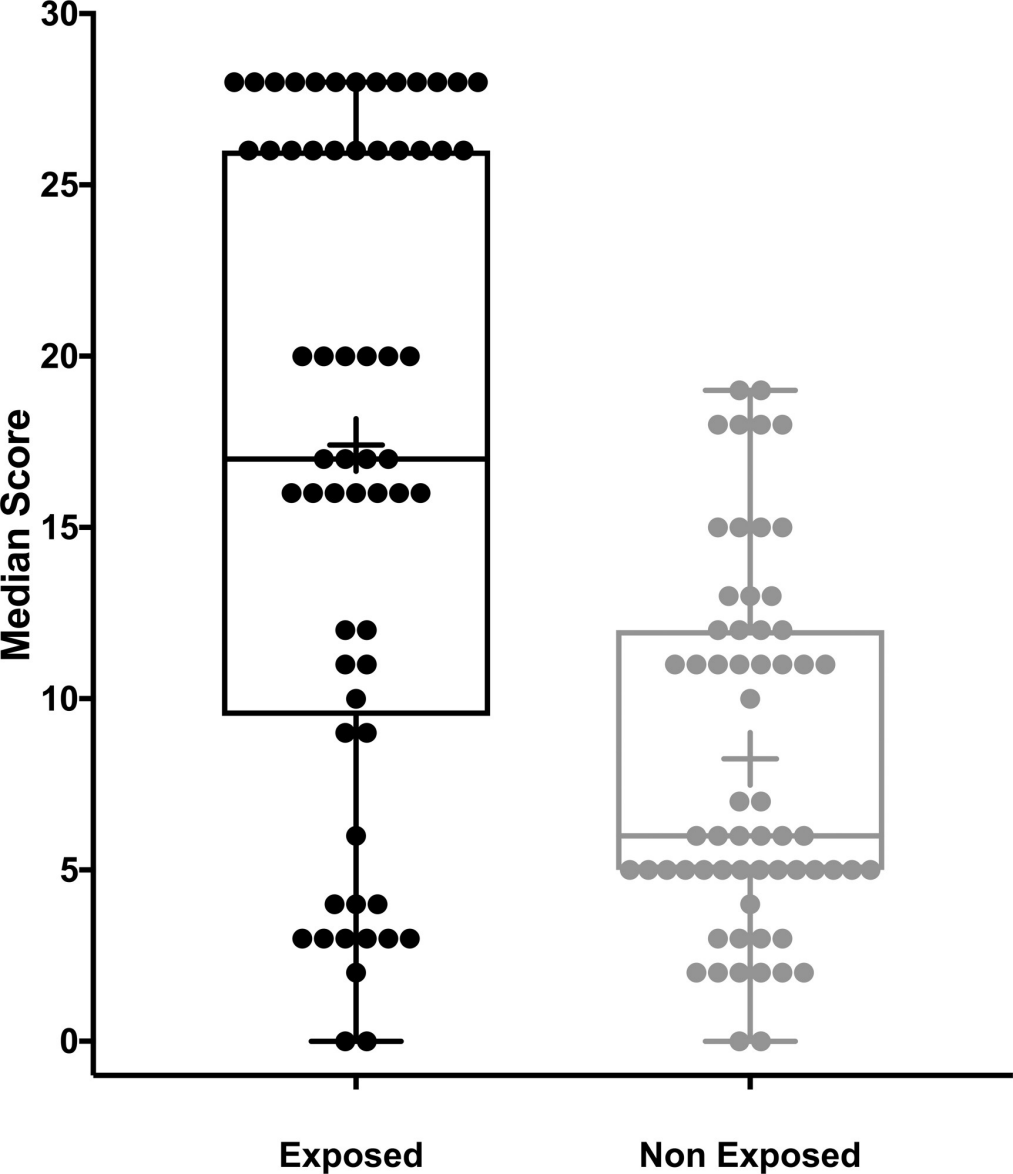
A box and whisker plot of the total score from the Donate questionnaire for exposed and non-exposed groups. Percentage of dry eye symptoms associates to the ocular Surface changes reported by the exposed and non- exposed groups to organic solvents used in the dry cleaning.

The results of the Donate questionnaire were determined for each observer and compared across groups that were exposed or not exposed to organic solvents. In general, 65% participants in the exposed group and the 28% of the non-exposed group obtained an overall score of more than 13, which indicated that dry eye symptoms were present in both groups (see Fig 1). Mann-Whitney U test showed that the median score of the exposed group was significantly higher than the non-exposed group (U = 53, *p*<0.0001). This indicated that overall, the exposed group reported more eye irritation symptoms than those not exposed to occupational levels of organic solvents.

As mentioned, the Donate Questionnaire comprised of 22 questions related to commonly reported eye irritation symptoms. A Chi squared analysis indicated that the exposed group reported significantly higher incidences of sandy sensation ((X^2^ (1, N = 124) = 235.91, *p* <0.005); tearing eyes sensation ((X^2^ (1, N = 124) = 234.67, *p* <0.005); foreign body sensation ((X^2^ (1, N = 124) = 217.12, *p*<0.005); tearing ((X^2^ (1, N = 124) = 173.44, *p*<0.005)); dry eye((X^2^ (1, N = 124) = 72.90, *p*<0.005); dryness ((X^2^ (1, N = 124) = 16.88, *p*<0.005); eyestrain ((X^2^ (1, N = 124) = 32.33, *p*<0.005); and heavy eyelids ((X^2^ (1, N = 124) = 5.66, *p*<0.005). But the following symptoms (p>0.05) were not: red eye; eye discharge; swollen lids; crusts and scales on lids; sticky eyes; sticky eyes when woke up; burning sensation; itching; eye strain; watery eyes; light sensitivity; blurred vision; eye fatigue; heavy eyelids and pain. As each symptom was rated on a Likert scale, we were able to determine whether those exposed to organic solvents reported greater frequency/severity in symptoms. We find that both groups differed with the dry eye group reporting greater severity only for symptoms relating to dry eye (U = 558, *p*<0.0001); sandy sensation (U = 101, *p*<0.0001); foreign body sensation (U = 75, *p*<0.0001), tearing (U = 78.5, *p*<0.0001); tearing eyes sensation (U = 31, *p*<0.0001); and dryness (U = 1342, *p*<0.0001). All other symptoms such as red eye (p = 0.16);swollen eyelids (p = 0.53); crusts and scales on lids (p>0.99); sticky eyes (p>0.99); eye discharge (p = 0.40); sticky eyes when woke up (p>0.99); burning sensation (p = 0.15); itching (p = 0.08); eye strain (p = 0.33); watery eyes (p = 0.13); light sensitivity (p = 0.11); blurred vision (p = 0.78); eye fatigue (p = 0.26); heavy eyelids (p = 0.08); pain (p>0.99) were not statistically significant between the two groups. In summary, individuals exposed to occupational levels of organic solvents report more and or greater severity of symptoms related to dry eyes and eye irritation.

### Quality and quantity of the tear film

**Fig 2:**
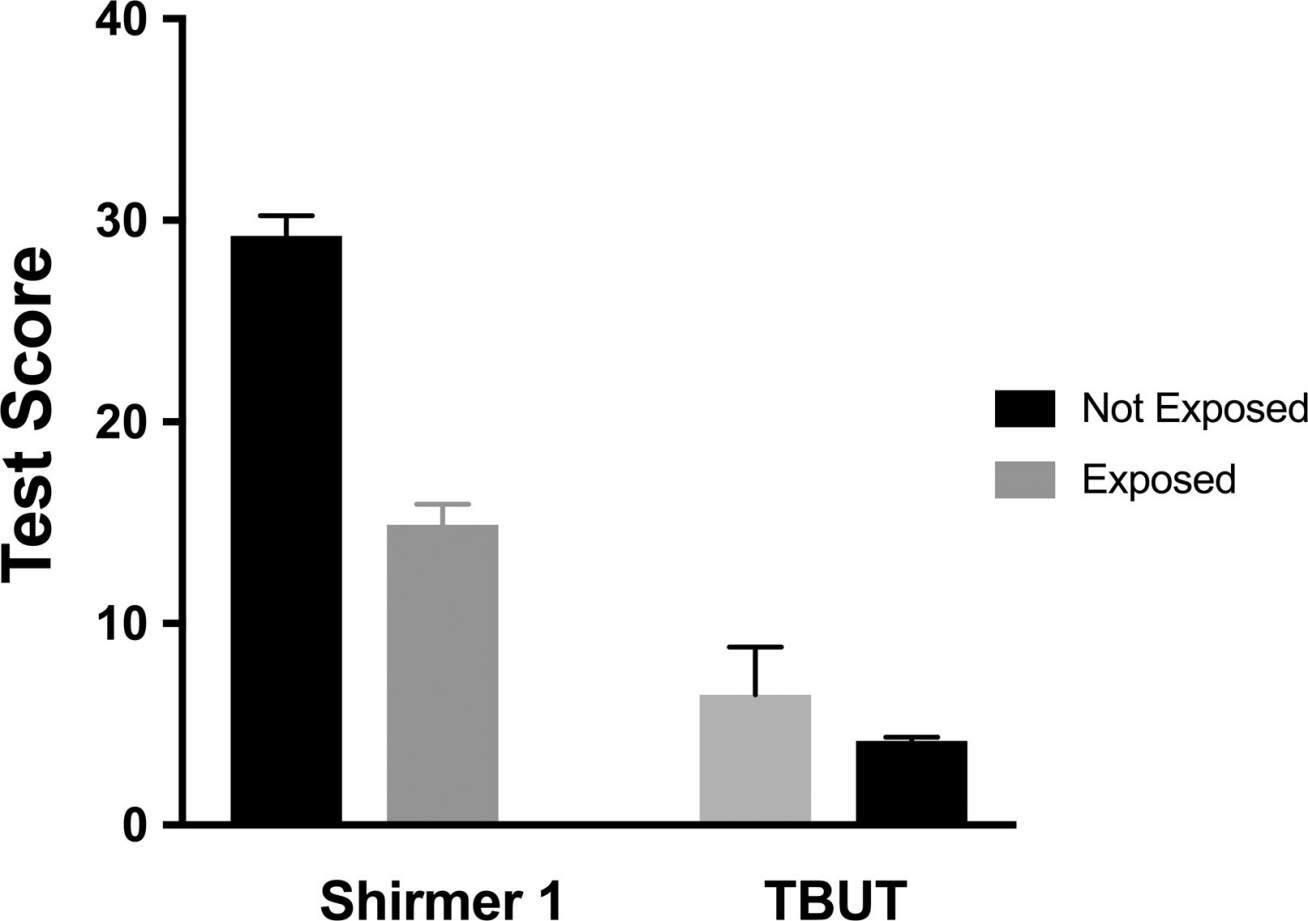
Average score (error bars signify 1 standard error of the mean) obtained in the Schirmer I test and TBUT in exposed and non-exposed participants to organic solvents used in the dry cleaning. The normal reference value for TBUT was greater than or equal to 5 seconds and for the Schirmer I test was 10mm/5min.

The amount of tear film measured by the Schirmer I test in the exposed group was 14.89 mm / 5 min (SD±8.08) and the TBUT was 4.17 sec (SD±1.42). In the non-exposed group, the Schirmer I test was 29.23 mm / 5 min (SD±7.96) and TBUT was 6.46 (SD±2.35). Statistical and clinical analysis showed that there was a significant difference between the two groups in these tests for quality (*t* 124 (+10.06), *p*<0,001), and quantity of the tear film (*t* 124 (-6.55), *p*<0,001); (Fig 2).

### Lipid interferometry

According to Guillón classification in the study, 94% of exposed group showed a wavy pattern and 6% marbling. This result shows that the tear lipid layer has a thickness about 50-70nm and the colour of the pattern was yellow gray. Considering that the thickness of the lipid layer in 96% of controls was 90-180nm and presented a yellow pattern, workers exposed to solvents presented a thinning lipid layer of the tear film and evaporation occurred more easily. Only the 6% of cases presented a marbleized pattern, denoting a clear thinning of the layer approximately 13-50nm, affecting the tear stability ((X^2^ (1, N = 124) = 0.44, *p <0*.*001*).

### Lissamine green staining

**Fig 3:**
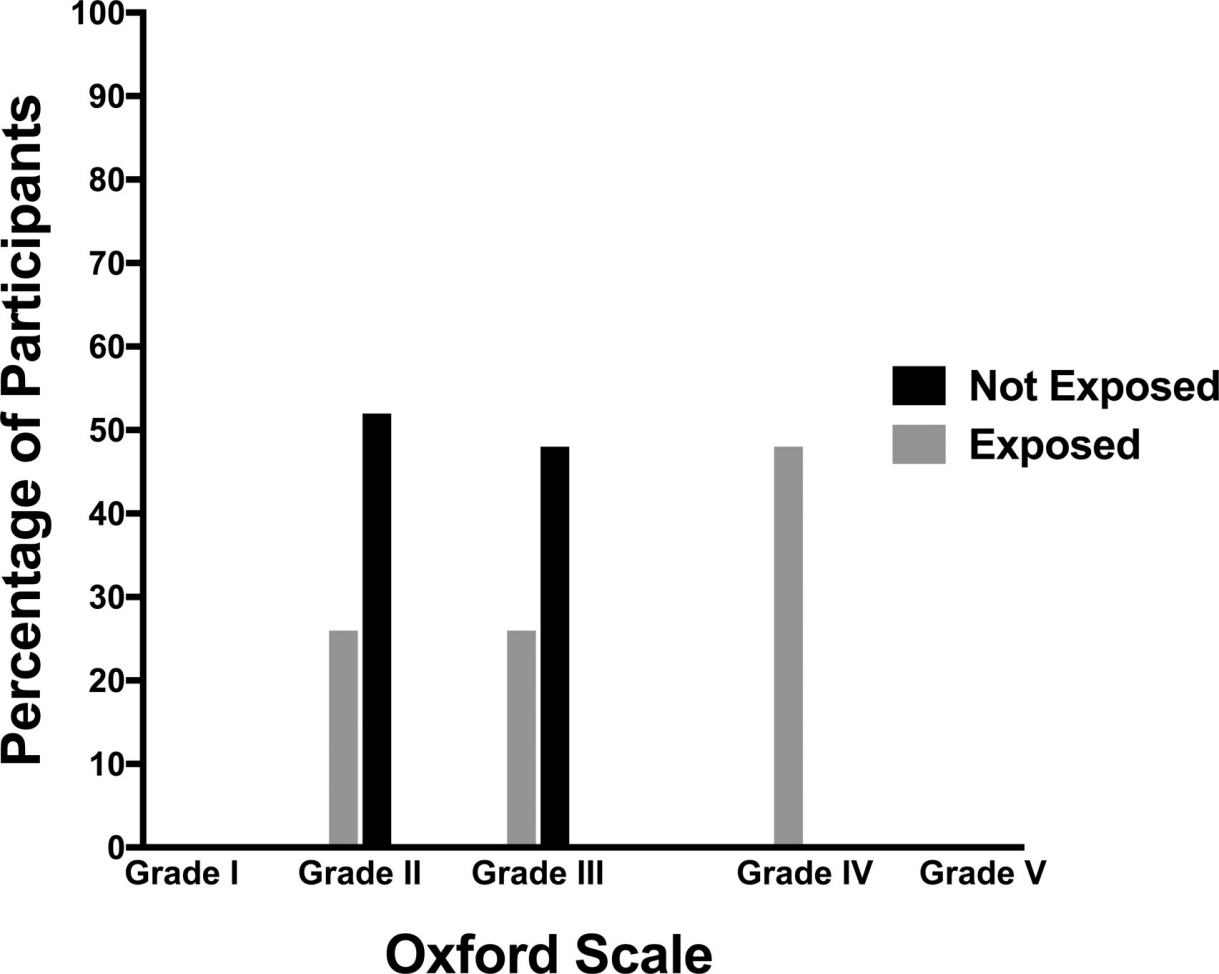
The percentage of participants with Lissamine green staining grades 0 to III according to the Oxford Scale, in exposed and non-exposed workers to organic solvents used in dry cleaning.

The exposed group showed that 26% of subjects had Lissamine green staining grade I, 26% grade II and 48% grade III. In contrast, 52% of the subjects in the non-exposed group presented grade 0 and none had grade II or III (Fig 3). There was a statistically significant difference between the groups (X^2^ (1, N = 124 = 80.96, *p* <0.0001).

### Conjunctival impression cytology

**Fig 4:**
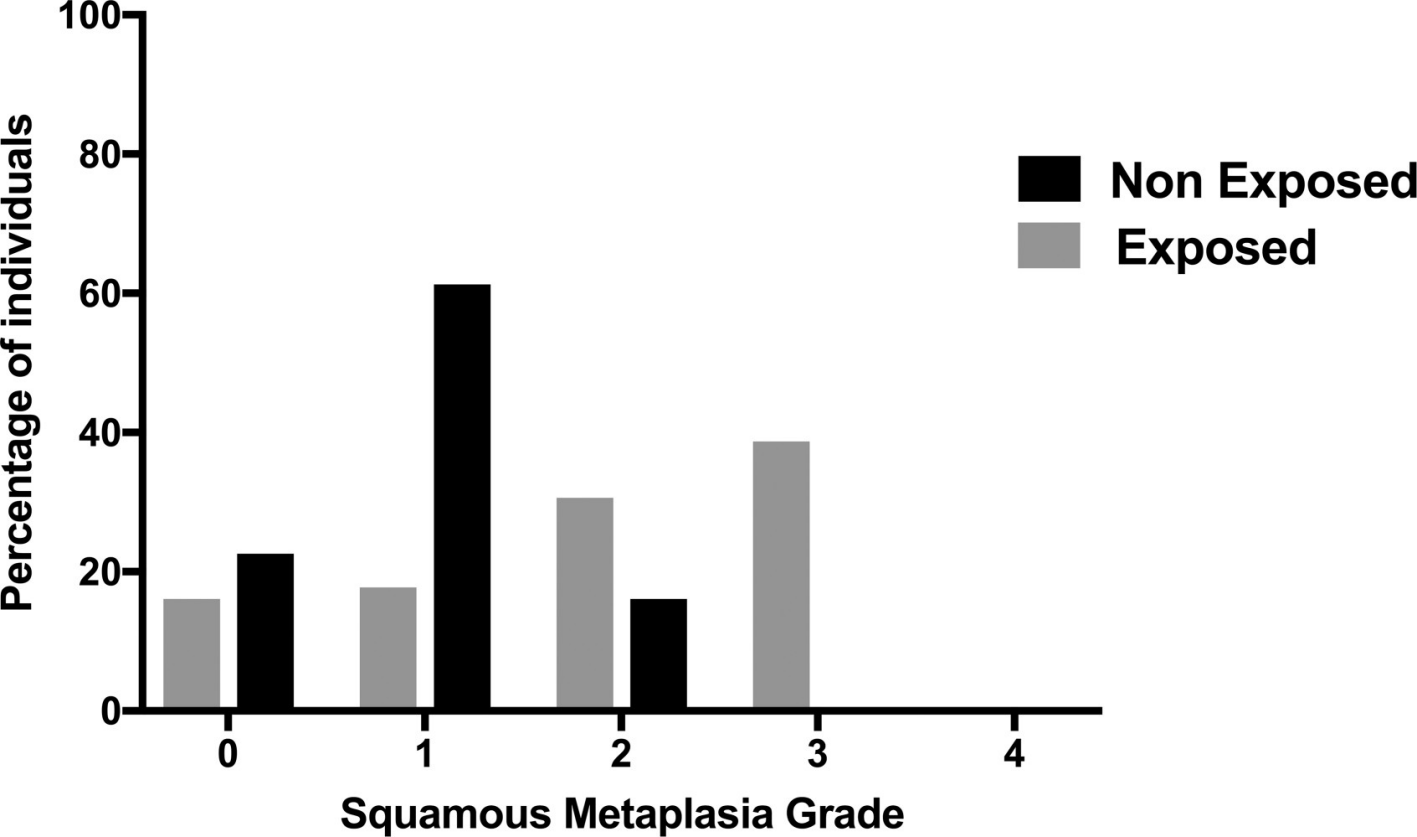
The percentage of participants with the different grades of squamous Metaplasia in exposed and non-exposed workers to organic solvents used in dry cleaning.

32.8% of participants in the exposed group had no squamous metaplasia grade (Grades 0 and 1); the 29.69% grade 2 and the 37.5% mild grade 3 or moderate. In the non-exposed group, 83.87% of cases had no squamous metaplasia grade (grade 0 and 1) and the 16.1% grade 2 or mild (Fig 4). There were statistically significant differences between groups (X^2^ (1, N = 124 = 115.3, *P* <0.0001).

### Correlations between Donate questionnaire and clinical tests

We also examined whether there was a relation between the severity of dry eye symptoms as indicated by the Donate questionnaire score and the clinical tests conducted in the present study. However, there was no correlation (Spearman’s Rho) for the Schirmer (exposed group rho = 0.19, p = 0.12; non-exposed group rho = -0.20, p 0.11), TBUT (exposed group rho = -0.06, p = 0.64; non-exposed rho = 0.19, p = 0.12), Impression Cytology (exposed group rho = -0.02, p = 0.83; non-exposed rho = -0.20, p = 0.1135) and Lissamine Green Staining (exposed group rho = -0.02, p = 0.86; non-exposed rho = -0.11 p = 0.35) with the Donate questionnaire. In addition, no correlation was observed between the outcomes of the Donate, Schirmer and TBUT and with the years of exposure (see Table 3).

**Table 3:** Correlations between objective and subjective test for diagnosis of dry eye and irritation.

	Exposed group		Non-exposed group	
Correlation	r / rho	p value	r / rho	p value
Exposure time/ Donate questionnaire	rho = -0.05	0.97	*N/A	*N/A
Exposure time/TBUT	r = -0.007	0.95	*N/A	*N/A
Exposure time/ Shirmer	r = -0.040	0.75	*N/A	*N/A
Donate Questionnaire/ Shirmer	rho = -0.19	0.12	rho = -0.20	0.11
Donate Questionnaire/TBUT	rho = -0.06	0.64	rho = 0.19	0.12
Donate Questionnaire/ Conjunctival Impression Cythology	rho = -0.02	0.83	rho = -0.20	0.11
Donate Questionnaire/Lissamine Green Staining	rho = -0.02	0.86	rho = -0.11	0.35

*Non-Applicable

## Discussion

The aim of this research was to identify if there was an association between ocular surface and tear film changes symptoms (and their severity) as measured using the Donate symptoms questionnaire. We found changes and damage to the ocular surface (Lissamine green staining and Impression Cytology), a decrease in the amount and quality of the tear film (Schirmer I and TBUT respectively) in workers exposed to occupational levels of organic solvents used in the dry cleaning industry, and this is associated with more symptoms of ocular irritation. In order to show whether the lipid layer of the tear film was affected, lipid interferometry was performed which found that 94% of cases exhibited a wavy pattern and 6% marbling, which suggests a significant thinning of this layer between 50-70nm. These results collectively suggest that chronic exposure to organic solvents is associated with significant changes to the ocular surface and more dry eye symptoms.

It is important to note that the dry-cleaning workplace is also contaminated with particulate matter from the items of clothing, and external stimuli such as cotton, wool, polyester, animal down in the air can also activate sensory receptors to increase dry -eye symptomatology in dry-cleaning workers. The concentration of such particulates was not measured in the present study, as it was beyond its scope, and we are unaware of any study that has quantified the type and levels of external stimuli in the dry-cleaning environment. However, we acknowledge that future studies are needed to investigate the concentration levels of certain particulates, and in addition to organic solvents, whether and how they contribute to dry-eye symptomatology. Finally, it is worth noting that our findings are similar to previous studies that have noted significant decrease in TBUT and ocular surface symptoms in painters exposed to organic solvents [10], who work in environments that have minimal garment particulates and or constant high temperatures. Importantly, this corroborating evidence suggests that organic solvents are perhaps the main cause of changes to the ocular surface and symptoms.

The lipid layer of the tear film is vital to protect the ocular surface from drying, the decrease in the thickness of the tear film (<70nm) is according to the presence of the dry eye condition classify as evaporative [29]. The main function of the lipid layer is to reduce the evaporation of the underlying aqueous phase when the evaporation rates increase, this could be to induce tear hyperosmolality, and this causes epithelial lesions on the ocular surface, a decrease in goblet cells, intercellular junctions, and disruption of the cell membranes, causing squamous metaplasia [16]. These findings are evidence microscopically by conjunctival impression cytology, a widely used technique for the diagnosis of the dry eye regardless of their origin [33]. More than 60% of the dry-cleaning workers in this study had squamous metaplasia similar to the results obtained *in vivo* with the Lissamine green staining, where the 74% of organic solvents exposed subjects presented mild to moderate degrees of staining.

The ocular surface epithelium damage might be caused by the alteration in the stability of the tear film by the organic solvents, or by direct interaction of the chemical on the cells of the ocular surface. *In vitro* studies have shown that the organic solvents cause disruption of the membranes and changes in their permeability, which is evidenced by the lack of cell viability and correlates with the lipophilicity of the compound [34]. *In vivo* studies conducted by Draize in rabbit eyes based on QSAR analysis (Quantitative structure-activity relationship) have shown that organic solvents can cause eye irritation due to their ability to solubilize the lipids of the cell membranes and attachment to proteins [35,36], which in this study might be the cause of the symptoms in dry cleaners. 65% of dry cleaners reported typical symptoms associated with dry eyes such as sandy sensation, foreign body sensations, and red eye. Previous studies had reported that individuals who use organic solvents at work suffer from dry eye and sandy sensation [4,17], which is similar to that reported in the present study.

The incidence of symptoms involves the activation of the sensory nerves of the ocular surface, due to several mechanisms. The reduction of the TBUT and the thinning of the tear film could cause the increment of the ocular discomfort [15], due to the tear evaporation. The cornea detects that need for more tears, to protect the epithelium and actives the cold corneal thermoreceptors (TRMP8) that are highly sensitive to dynamic downward shifts in temperature, such as occur during tear evaporation, and their primary function appears to be to control the basal tear secretion [37]. When there is an epithelial damage, the nerve response in the cornea is increased by the local inflammatory response. When the corneal nerves are activated, they release neuropeptides, which contribute to the inflammatory reaction. These are sensitized by mediators such as prostaglandins, histamine or bradykinin, which may cause spontaneous activity on the trigeminal nociceptors causing low pain threshold and increase the response to a new stimulus leading to pain and hyperalgesia [38].

Interestingly, in the present study the relationship between symptoms (as reported on the Donate questionnaire) and clinical laboratory tests were not statistically significant. This was not surprising given that previous studies have also noted the poor correlations between symptoms assessed using questionnaires and clinical findings in patients with dry eye. For example, applying the Dry Eye Questionnaire, a small correlation has been reported between the symptoms and cornea staining fluorescein staining, Lissamine green staining, Schirmer's test and TBUT (r <0.22–0.45) [39]. Similarly, with the Ocular Surface Disease Index (OSDI), a very low correlation has been reported (r <0.14), between symptoms and osmolarity, TBUT, Schirmer's test, Meibomian gland dysfunction and corneal staining fluorescein [40,41]. However, others have found an association between symptoms and clinical parameters such as Schirmer test and TBUT [15,24], as well as a good correlation when comparing the total score of the questionnaire with the disease severity degree [15,42] and the majority of these studies have shown that dry eye patients have greater symptomatology than subjects without the disease. It seems that several factors influence the lack of correlation between the symptoms and clinical signs, such as the type of questionnaire, the repeatability, and reproducibility of the clinical tests, the degree of clinical severity [19,41,43] and of course the self-perception of the patient's health [41]. Particularly for the latter, subjective response and tolerance to pain and irritation are known to significantly vary between individuals, and this might be a contributing factor on the reporting of the severity of symptoms.

Additionally, we find no correlation between years of exposure and the Donate questionnaire, Schirmer and TBUT (see Table 3). These findings suggest that the possible effects of organic solvents on the ocular surface are unlikely to be progressive over time, but rather occur once workers are exposed to them, though the severity of the symptoms might vary due to individual differences. Additionally, it is possible that the effect of organic solvent on dry eye symptoms might be short-lived and recovery occurs quickly after exposure has stopped. Accordingly, it is possible that recency of exposure might offer a more valid measure than the conventional cumulative years of exposure. Future studies may wish to investigate whether and how long dry eye symptoms and the ocular surface recover after the cessation of exposure to organic solvents.

In our case, we speculate that the symptomatology was directly stimulated by the organic solvent’s exposure. Volatile organic compounds can interact with one or more sensory neurons receptors, therefore, could elucidate various types of responses, they stimulate the trigeminal somatosensory system by direct activation of sensory neurons, or indirectly by inflammatory mediators that were generated by the chemical in the injured tissue [21,44]

Chemicals can directly activate polymodal nociceptors, inducing burning, stinging, irritation or pain in the eyes and upper airways [6,45]. Cytokines, inflammatory mediators and chemicals use the same receptors on trigeminal nerve, TRPA1 channels are most associated with the direct response to many volatile organic compounds and TRPV1 to the endogenous agents released during the inflammatory response [8]. Their function has been related to the translation of pain in inflammation and as exteroceptive sentinels of chemesthesis [7,9,46].

The findings of the present study suggest that organic solvents might activate the nociceptors causing eye discomfort and irritation, but further investigation is needed to measure and establish the relationship between functional (e.g., corneal sensitivity) and structural (i.e., corneal nerve morphology) changes to the eye from organic solvent exposure. Such an investigation is of importance as the present study is cross sectional in design statements regarding cause and effect cannot be immediately made, and further studies are needed to empirically quantify the chemical action of organic solvents on changing the tear surface, corneal sensitivity, corneal nerve morphology and reported dry eye and eye irritation symptoms. Though a causal link has already been established in animal models [35,36] and suggested in our previous work [14, 15, 16].

## Conclusion

Workers in dry cleaning establishments have changes in the tear film lipid layer that possibly causes evaporative dry eye with the signs and symptoms that characterize this disease. More studies are needed to determine the type of activation generated by the organic solvents on the trigeminal and if this is connected to the neurological changes reported.

## Supporting information

S1 Table Symptoms donate analysis OS. Statistical correlations and difference between groups.
(PZFX)

S2 Table Data ocular surface. Database with average calculations.
(XLSX)
